# Diagnostic Challenges of Aleukemic Myeloid Sarcoma

**DOI:** 10.7759/cureus.64644

**Published:** 2024-07-16

**Authors:** Nathanial W Hansen, Shelby Boock

**Affiliations:** 1 Dermatology, Corewell Health, Farmington Hills, USA; 2 Dermatology, Philadelphia College of Osteopathic Medicine, Philadelphia, USA

**Keywords:** chloroma, aleukemic myeloid sarcoma, non-leukemic myeloid sarcoma, myeloid sarcoma, aleukemic

## Abstract

Myeloid sarcoma (MS) represents a neoplastic proliferation characterized by immature myeloid precursor cells. Among its variants, aleukemic MS is an uncommon subtype, manifesting as skin involvement sparing the peripheral blood or bone marrow. The non-specific cutaneous presentation coupled with the lack of associated symptoms poses a diagnostic challenge for providers. In this report, we present a case of an 83-year-old woman who presented with violaceous nodules located in the center of her right shin. A biopsy of the lesion unveiled a diagnosis of MS, yet notably lacked peripheral blood involvement. Three months after the initial diagnosis, the MS was found in the common bile duct, still without bone marrow involvement. With a relatively poor prognosis, the rapid diagnosis and treatment of MS are crucial.

## Introduction

Myeloid sarcoma (MS), an extramedullary manifestation of acute myeloid leukemia (AML), myeloproliferative neoplasms, or myelodysplastic syndromes, is composed of a proliferation of immature myeloid precursor cells. On rare occasions, MS may manifest as a solely cutaneous entity lacking any peripheral blood or bone marrow involvement. This is classified as aleukemic or non-leukemic MS. This challenging presentation occurs in 2.2% of patients diagnosed with AML [1]. While AML primarily affects bone marrow, aleukemic MS commonly infiltrates the skin, soft tissues, lymph nodes, testis, and mammary glands [2]. In cases of isolated skin involvement, the question remains whether the cancerous cells originate in the skin or bone marrow [3].

Due to a lack of defining cutaneous features, the differential diagnoses can be extensive, especially when there is a lack of previous medical history [4]. Therefore, diagnosis relies heavily on biopsies and immunohistochemical staining for confirmation [3]. Once a diagnosis is confirmed, prompt initiation of treatment is imperative. Chemotherapy stands as the most effective treatment, as surgical excision and radiation have not shown efficacy in halting progression [5].

We present a case of an 83-year-old female patient with cutaneous aleukemic myeloid sarcoma.

## Case presentation

**Figure 1:**
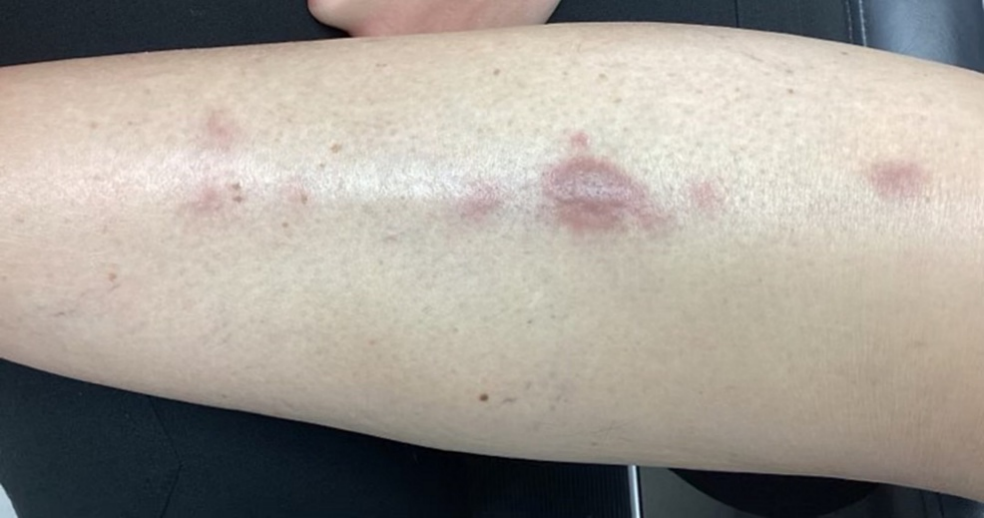
Lesions on presentation

**Figure 2:**
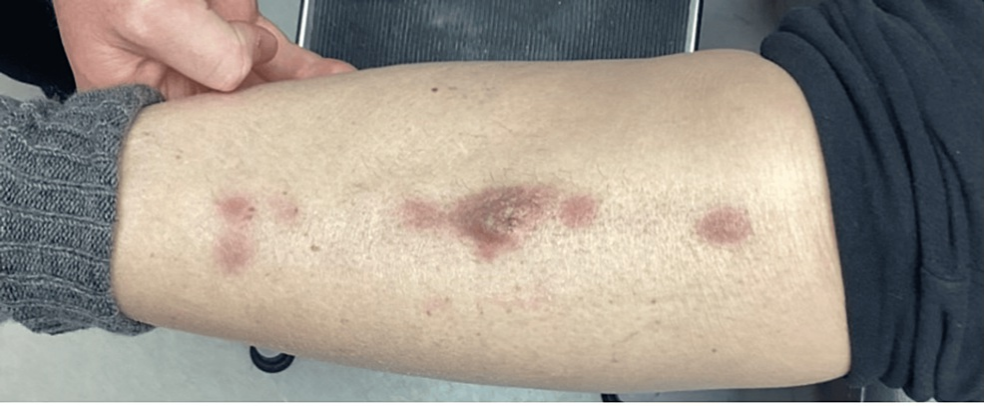
Lesions eight weeks following the initial presentation

The patient was an 83-year-old female with a past medical history of hypertension, dyslipidemia, and endometriosis who presented with “red bumps” on her right anterior shin that she first noticed six weeks prior (Figures 1, 2). 

**Figure 3:**
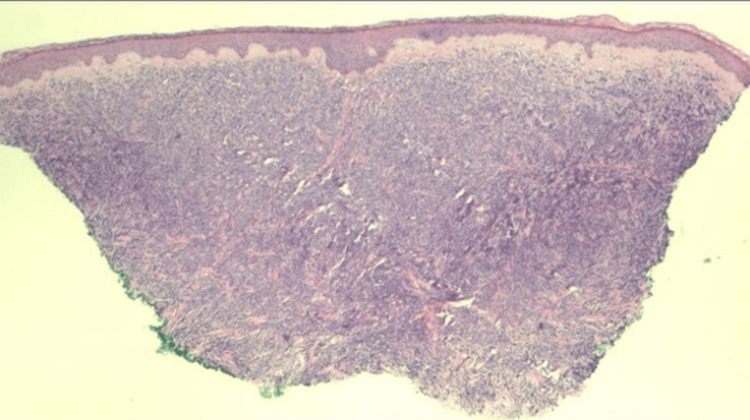
2x Magnification with hematoxylin and eosin (H&E) stain showing a dermal infiltrate of sheets of mononuclear lymphoid cells

**Figure 4:**
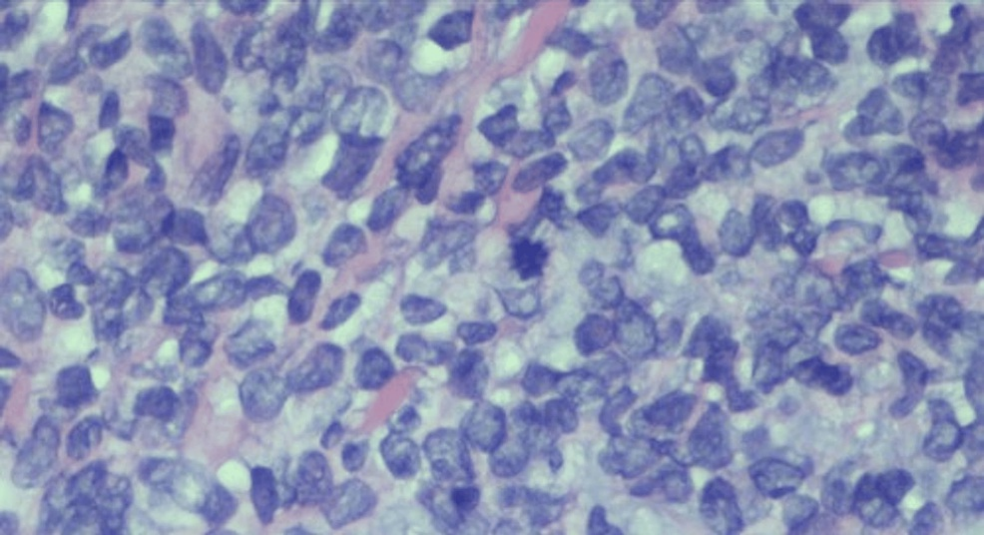
40x magnification with hematoxylin and eosin (H&E) stain showing cells with dispersed chromatin with a high nuclear-to-cytoplasmic ratio and mitotic figures

The lesions had been slowly enlarging, and she denied pruritus and tenderness on palpation. She had no personal or family history of similar lesions or skin cancers. She denied fever, cough, night sweats, shortness of breath, gastrointestinal changes, dysuria, myalgia, and arthralgias but stated she lost 10 pounds over the past year. On physical exam, there were five linear, violaceous nodules located anteriorly in the center of the right shin. The largest lesion measured 1.5 cm x 0.6 cm. The nodules were not tender to palpation and displayed no secondary changes. A punch biopsy of the largest central lesion showed a “Malignant Hematolymphoid Neoplasm.” The pathology showed a diffuse, dermal sheet growth pattern of lymphocytic infiltrates that extended to the deep dermis sparing the epidermis predominantly composed of large cells. Immunohistochemical staining was diffusely positive for CD45 (LCA) and BCL-2, and negative for CD3, CD20, CD10, BCL-6, CD330, CK7, CK20, AE1/AE3, and SOX-10 (Figures 3, 4).

The patient was then referred to hematology and oncology for further workup. Flow cytometry was positive for CD43, Ki-67, c-myc, CD117, myeloperoxidase, and lysozyme and negative for B-cell and T-cell markers. She was seen by hematology and underwent a bone marrow biopsy and an AML fluorescence in situ hybridization (FISH) panel. Results for both tests were negative for AML. The chosen course of treatment for this patient was decitabine and venetoclax. On the third day of treatment, the patient appeared jaundiced and had elevated liver function tests. She was admitted to the hospital for acute cholecystitis. During her treatment, she was continued on decitabine. She was given Zosyn and underwent a magnetic resonance cholangiopancreatography (MRCP) that showed areas of biliary stricturing and dilation notably worrisome for malignancy. During endoscopic retrograde cholangiopancreatography (ERCP), a mass was found in the lower third of the common bile duct. Fine-needle aspiration was sent for biopsy and cytology and a stent was placed. Biopsy revealed sheets of medium-sized mononuclear cells that are positive for CD117, myeloperoxidase, and negative for B-cell and T-cell markers. These findings were consistent with the patient’s already diagnosed myeloid sarcoma. To date, she has completed one cycle of decitabine and is currently being maintained on venetoclax.

## Discussion

To establish a confirmed diagnosis of aleukemic MS, specific criteria must be met. Pathological confirmation via biopsy is necessary, peripheral blood or bone marrow testing shows an absence of leukemic cells, and no previous history of AML, myeloproliferative neoplasms, or myelodysplastic syndromes was present [1].

A retrospective study revealed a median age of diagnosis at 37 years old, with females being 1.9 times more likely to be affected [1]. In comparison to prior cases of cutaneous MS, this patient presented at an age almost 50 years older than the average age of presentation [1]. Typical presenting symptoms include masses, nodules, local bleeding, numbness, or pain [1]. Lesions have been described as red-brown plaques, pink-brown-violaceous papules, and nodules [3,6]. Notably, the patient in this case did not exhibit these commonly reported symptoms. The absence of symptoms and concerning visible features may have delayed the patient from receiving earlier treatment.

A previous study identified CD43, lysozyme, myeloperoxidase (MPO), CD68 (or CD163), CD117, CD3, and CD20 as markers for the majority of MS variants [7]. Out of the seven markers, our patient’s lower limb lesion tested positive for CD43, CD117, myeloperoxidase, and lysozyme. Additionally, her biliary duct stricture was positive for markers CD117 and myeloperoxidase.

Early diagnosis and prompt initiation of treatment are crucial for these patients. Currently, there is no standardized, effective chemotherapy regimen used for aleukemic MS. Various combinations of cytarabine, idarubicin, mitoxantrone, homoharringtonine, cyclophosphamide, vincristine, prednisone, adriamycin, and daunomycin have been attempted across several studies [1,8,9]. Using chemotherapy alone, the complete remission rate was 57.9%, however, the relapse and progression to AML rates remained high at 73.7% and 47.4%, respectively [1,10]. The prognosis is relatively short, with a median overall survival rate of 30 months [1,11].

Exploring aleukemic MS is relevant, as it delves into the intricate connection between hematological malignancies and dermatological manifestations. The road to diagnosis for patients with aleukemic MS is difficult, and delays in appropriate treatment can lead to devastating outcomes in these patients. Three months after the patient’s initial presentation with the lesion depicted in Figure 1, MS was also found in the common bile duct. Cutaneous myeloid sarcoma without bone marrow involvement is uncommon, and it is even more uncommon to have multiple organ systems affected with no signs of bone marrow involvement or AML. Physicians should be aware of the need for prompt workup and referral to the appropriate specialists.

## Conclusions

Myeloid sarcoma is an extramedullary manifestation of acute myeloid leukemia myeloproliferative neoplasms or myelodysplastic syndromes and rarely may manifest as a solely cutaneous entity lacking any peripheral blood or bone marrow involvement. Due to a lack of defining cutaneous features, diagnosis of this condition can be difficult and a high index of suspicion is required. Comprehensive clinical and pathological evaluation of the skin and involved organ systems is vital to diagnose this uncommon condition, and a multi-system workup is required to begin prompt treatment.
